# Association Between Trajectory Patterns of Body Mass Index Change Up to 10 Months and Early Gut Microbiota in Preterm Infants

**DOI:** 10.3389/fmicb.2022.828275

**Published:** 2022-04-27

**Authors:** Jun Qiu, Changci Zhou, Shiting Xiang, Jie Dong, Qifeng Zhu, Jieyun Yin, Xiulan Lu, Zhenghui Xiao

**Affiliations:** ^1^Pediatrics Research Institute of Hunan Province, Hunan Children’s Hospital, Changsha, China; ^2^Academy of Pediatrics, Hengyang Medical School, University of South China, Hengyang, China; ^3^School of Public Health, Medical College of Soochow University, Suzhou, China; ^4^Department of Intensive Care Unit, Hunan Children’s Hospital, Changsha, China

**Keywords:** preterm infants, gut microbiota, BMI growth trajectory, obesity, 16S rDNA gene sequencing

## Abstract

Recent research suggests that gut microbiota plays an important role in the occurrence and development of excessive weight and obesity, and the early-life gut microbiota may be correlated with weight gain and later growth. However, the association between neonatal gut microbiota, particularly in preterm infants, and excessive weight and obesity remains unclear. To evaluate the relationship between gut microbiota and body mass index (BMI) growth trajectories in preterm infants, we examined microbial composition by performing 16S rDNA gene sequencing on the fecal samples from 75 preterm infants within 3 months after birth who were hospitalized in the neonatal intensive care unit of Hunan Children’s Hospital from August 1, 2018 to October 31, 2019. Then, we collected their physical growth information during 0–10 months. Latent growth mixture models were used to estimate growth trajectories of infantile BMI, and the relationship between the gut microbiota and the BMI growth trajectories was analyzed. The results demonstrated that there were 63,305 and 61 operational taxonomic units in the higher BMI group (*n* = 18), the lower BMI group (*n* = 51), and the BMI catch-up group (*n* = 6), respectively. There were significant differences in the abundance of the gut microbiota, but no significant differences in the diversity of it between the lower and the higher BMI group. The BMI growth trajectories could not be clearly distinguished because principal component analysis showed that gut microbiota composition among these three groups was similar. The three groups were dominated by *Firmicutes* and *Proteobacteria* in gut microbiota composition, and the abundance of *Lactobacillus* in the higher BMI group was significantly different from the lower BMI group. Further intervention experiments and dynamic monitoring are needed to determine the causal relationship between gut microbiota differences and the BMI change.

## Introduction

Excessive weight and obesity are important clinical and public health problems worldwide (Kelly et al., 2008). From 1975 to 2016, the global prevalence of excessive weight and obesity in children aged 5–19 years increased sharply, from 0.7 to 5.6% for girls and 0.9 to 7.8% for boys, respectively [NCD Risk Factor Collaboration (NCD-RisC), 2017]. The rate of excessive weight and obesity among children and adolescents in China is increasing as well (He et al., 2017). Research has shown that the excessive weight rate among children and adolescents in China aged 6–17 years increased from 4.27% in 1991 to 11.70% in 2015, whereas the obesity rate increased from 2.41 to 12.74% during the same period (Ma et al., 2020). Excessive weight and obesity not only directly affect children’s physical development and physiological function and impair their motor and learning ability (Warschburger, 2005), but also increase the risk of coronary heart disease, hypertension, diabetes, and tumors in their adulthood (Zhang and Ma, 2017). Excessive weight and obesity bring huge mental and economic burdens to these children, their families, and the society (Kelly et al., 2008).

It is generally believed that obesity is caused by an imbalance between energy intake and energy expenditure. A number of hereditary, behavioral, and environmental factors have contributed to obesity. Recently, many studies have highlighted the effect of gut microbiota in the occurrence and development of excessive weight and obesity. Chen et al. (2020) also demonstrated that infant antibiotic exposure was associated with disruption of the gut microbiota and the higher risks of childhood obesity and increased adiposity. Gut microbiota is composed of a variety of microorganisms colonized in the gastrointestinal tract of organisms, which has developed a potential outcome of symbiotic relationships (Hooper and Gordon, 2001). Gut microbiota plays an important role in human beings, such as regulating the immune response and promoting the absorption of nutrients. It is mainly composed of six phyla including *Firmicutes, Bacteroidetes, Actinobacteria, Fusobacteria, Proteobacteria*, and *Verrucomicrobia* (Eckburg et al., 2005; Koboziev et al., 2014). Previous studies investigated the effect of gut microbiota on the occurrence of obesity. They found that, in mice and even in children, the obese individual had a higher proportion of *Firmicutes* and a lower proportion of *Bacteroidetes* (Ley et al., 2005; Abdallah Ismail et al., 2011). Moreover, other studies noticed that the composition, quantity, and proportion of gut microbiota in obese children were distinctly different from those of normal ones (Gao and Wan, 2017). Research on the composition of gut microbiota may aid the prevention and treatment of child obesity (Sekirov et al., 2010).

At present, studies on the potential association between gut microbiota and obesity mostly focused on preschool and school-age children, and most of them are cross-sectional studies (Li et al., 2015; Hollister et al., 2018; Zhang et al., 2018). However, obesity in infants has been attracted more and more attention. It is demonstrated that maternal gut microbiota is correlated with the growth and development of newborns in their first 18 months (García-Mantrana et al., 2020). The gut microbiota composition in infancy is related to subsequent body mass index (BMI) in childhood (Korpela et al., 2017). Some studies have shown that preterm infants have a higher forward risk of diabetes and obesity-related diseases than full-term infants (Chinese Journal of Woman and Child Health Research, 2017).

However, there are no existing studies on the relationship between gut microbiota and obesity in preterm infants. BMI is the most widely adopted indicator of obesity, but BMI and changes in BMI during childhood are highly heterogeneous (Mattsson et al., 2019; Sun et al., 2021). Hence, a single cross-sectional BMI measurement ignores the dynamics of BMI and may be insufficient to assess pediatric obesity-related disease risk (Simmonds et al., 2015). Fortunately, BMI trajectory, based on the latent class growth model (LCGM), utilizing at least three repeated measures in a longitudinal cohort can provide additional information about BMI (Mattsson et al., 2019). A study identified distinct BMI trajectories during the first 2 years of infants and showed that infants in the middle and upper trajectory groups were more likely to suffer excessive weight/obesity (Zhang et al., 2021). Besides, many studies have confirmed that different BMI growth trajectories contribute to different risks of obesity, cardiovascular diseases, and metabolic syndrome (Rolland-Cachera et al., 1984; Braun et al., 2018). Therefore, in this study, we aimed at exploring the relationship between gut microbiota and BMI growth trajectories in preterm infants and hoping to provide etiological references, prevention, and treatments for childhood obesity. With this goal, fecal samples of neonates’ first defecation after hospitalization were collected and then analyzed. Infantile BMI was collected during 0–10 months, and latent growth mixture models (LCGMMs) were used to classify according to their different BMI growth trajectories.

## Materials and Methods

### Study Population

A total of 137 preterm infants hospitalized in the neonatal intensive care unit of Hunan Children’s Hospital from August 1, 2018 to October 31, 2019, were enrolled in this study. Inclusion criteria were as follows: (a) preterm infants vaginally delivered without asphyxia, intrauterine distress or amniotic fluid pollution at birth, and receiving exclusive breastfeeding after birth. The age at admission was more than 7 days and less than 60 days. (b) The maternal age was between 20 and 35 years, without bad habits such as drinking and smoking, special dietary habits, infection within 1 month before delivery, or pregnancy complications. (c) The preterm infants did not show the symptoms of vomiting, hematochezia, abdominal distension, and other gastrointestinal diseases within 1 week before admission. (d) The newborns’ legal guardians had signed informed consent form. Exclusion criteria were as follows: (a) Obesity was caused by medication, heredity, and endocrine disorder, and subjects with intestinal diseases, such as diarrhea, constipation, and gastroenteritis. (b) Subjects were treated with microecologics and antibiotics within 2 weeks prior to admission. The first fecal samples of the preterm infants after admission were collected. The height and weight of infants were assessed at the age of 3, 4, 6, 8, 9, and 10 months. A total of 75 participants were finally included in the study, after exclusion of 62 subjects who had fewer than three follow-up visits. The 75 preterm infants were divided into higher BMI group (*n* = 18), lower BMI group (*n* = 51), and BMI catch-up group (*n* = 6) according to the BMI growth trajectories.

This study was approved by the Ethics Committee of Hunan Children’s Hospital (no. HCHLL-2018-64), under the guidelines of the Declaration of Helsinki, and obtained the formal written informed consent from all the parents and guardians of the participating infants.

### Samples and General Information Collection

Fecal samples of neonates’ first defecation after hospitalization were collected and immediately frozen in the ice boxes. The samples were then transported to laboratory within 2 h and stored at −80°C. Questionnaires were sent to collect information about the maternal pregnancy status and neonatal birth information for neonates eligible for the inclusion criteria. The hospitalization data of neonates were extracted from the medical record system.

### Physical Development Measurement and Data Grouping

**FIGURE 1:**
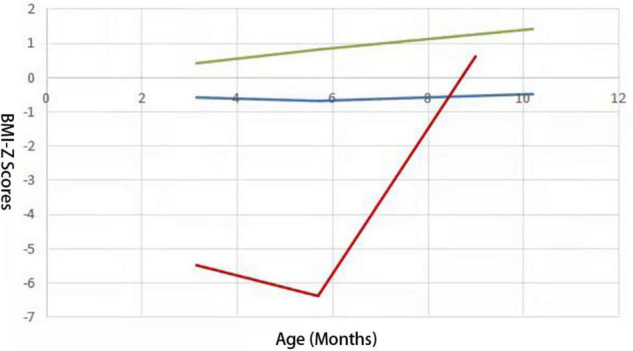
Three BMI growth trajectories from 75 preterm infants identified from LCGMM. Lines with different colors represent different trajectory groups. The abscissa represents BMI-*Z* scores and the ordinate represents the age (months). The blue one represents the lower BMI group; the green one represents the higher BMI group, and the red one represents the BMI catch-up group.

Follow-up was performed at 3, 4, 6, 8, 9, and 10 months of age using WHS-1 infant physical examination digital meter to measure subjects’ height and weight by trained professionals. According to the 2014 World Health Organization Child Physical Development Evaluation Standard (de Onis, 2015), age for BMI-*Z* score of every participant was calculated at each examination time point. LGMMs were used to identify latent growth trajectories of infantile BMI subjected to age, and the “PROC Traj” statement in SAS software was used to establish the models (Jones and Nagin, 2007). The fit indices for one- to four-class LGMM for BMI are presented in Table 1. Based on a comprehensive comparison over fit indices and the prior knowledge about BMI-*Z* score trajectory in childhood and adolescence (Marioni et al., 2014), we finally selected three trajectories and took the trajectories with polynomial orders 0, 1, and 3 as the optimal models, respectively, which were divided into BMI catch-up group, lower BMI group, and higher BMI group (Figure 1).

**TABLE 1:** Model parameters for different classifications.

Group	Maximum BIC	Mean PP	Percentage of AvePP > 0.7 (%)	Percentage of persons in each group (%)
1	−362.61	1.00	100.0	100.0
2	−351.38	0.94/0.99	83.3/100.0	7.7/92.3
3	−352.01	0.89/0.76/0.92	80.4/72.2/83.3	66.7/25.8/7.5
4	−350.84	1.00/0.91/0.85/1.00	100.0/87.2/81.8/100.0	1.4/61.4/30.2/7.0

*BIC, Bayesian information criterion; AIC, Akaike information criterion; AvePP, average trajectory posterior probability.*

### DNA Extraction and High-Throughput 16S rDNA Gene Sequencing

Total DNA was isolated from all fecal samples by QIAamp DNA Stool Mini Kit. The isolated DNA was then used as a template for the amplification of the V4 + V5 region of 16s rDNA gene using the 515F/907R (515F: 5′-GTGCCAGCMGCCGCGG-3′/907R: 5′-CCGTCAATTCMTTTRAGTTT-3′) primer for polymerase chain reaction. Amplicons were extracted from 2% agarose gels, purified using the AxyPrep DNA Gel Extraction Kit, quantified using QuantiFluorTM-ST, and sequenced on the Illumina MiSeq platform.

### Gut Microbial Analysis

The raw reads were further filtered according to the following rules to improve the accuracy of later analysis: (1) removing reads containing bases with terminal quality less than 20 and sequences with length less than 100 bp using TrimGalore software. (2) Merge sequences were obtained by splicing pairs of sequences using FLASH2 software, and low-quality sequences were removed. (3) Removing the primers using mothur software. (4) Sequences with base mismatch rate of more than 2% and length less than 100 bp were removed using the usearch, and the optimized sequences with high-quality and credibility were obtained.

The filtered sequences were clustered into operational taxonomic units (OTUs) of ≥97% similarity, and the sequence with the highest abundance was considered a representative sequence within each cluster.

α diversity was evaluated using abundance indices (Chao1 and ACE) and diversity indices (Shannon and Simpson). Curve analysis including rarefaction curves and Shannon–Wiener curves was used to reflect the rationality of sample size. Venn diagram based on OTU abundance was used to show the richness and similarity of microbiota composition among groups. Principal component analysis (PCA) based on OTU abundance table was used to evaluate community composition and structure of microbiota.

Metastats analysis was performed to compare samples among groups, and the species statistically significant among the groups at each classification level were discovered. *p* < 0.05 was considered to be statistically significant.

### Statistical Analysis

The experimental data were analyzed by SAS 9.2 and R statistical software. Differences in quantitative data were assessed by analysis of variance, differences in categorical data were evaluated by χ^2^ test, and differences in diversity indices of gut microbiota were evaluated by rank-sum test. *p* < 0.05 was considered to be statistically significant.

## Results

### Characteristics of the Study Population

The characteristics of 75 preterm infants are summarized in Table 2. The 75 preterm infants were divided into higher BMI group (*n* = 18), lower BMI group (*n* = 51), and BMI catch-up group (*n* = 6). There were no differences in gender, sampling age, birth weight, gestational age, and the prevalence of pneumonia and sepsis.

**TABLE 2:** Comparisons of the characteristics of preterm infants’ information among the three groups.

	BMI catch-up group (*n* = 6)	Lower BMI group (*n* = 51)	Higher BMI group (*n* = 18)	χ^2^/*F*	*p*
Male, n (%)	5 (83.3)/1 (16.7)	30 (58.8)/21 (41.2)	11 (61.1)/7 (37.9)	1.00	0.317
Sampling age (days)	27.00 ± 19.00	34.44 ± 17.11	40.00 ± 21.97	0.51	0.605
Birth weight (kg)	1.36 ± 0.42	1.96 ± 0.51	1.78 ± 0.41	2.05	0.151
Gestational age (weeks)	33.67 ± 1.15	32.00 ± 2.77	31.00 ± 4.15	0.78	0.468
Pneumonia, n (%)	1 (16.7)/5 (83.3)	10 (19.6)/41 (80.4)	2 (11.1)/16 (88.9)	0.13	0.722
Sepsis, n (%)	1 (16.7)/5 (83.3)	2 (3.9)/49 (96.1)	1 (5.6)/17 (94.4)	0.73	0.394

### Microbial Diversity Analysis

#### Curve Analysis

**FIGURE 2:**
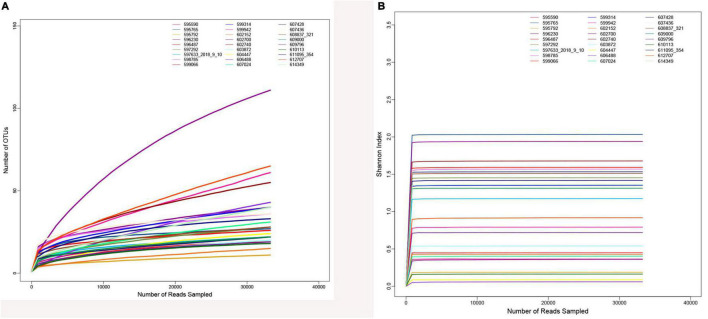
Sample curve analysis. **(A)** Sample rarefaction curves. **(B)** Sample Shannon–Wiener curves.

After quality control and filtering of raw sequences, there were 3,088,914 optimized sequences in total, with a mean of 114,404.22 per sample. The rarefaction curve (Figure 2A) and the Shannon–Wiener curve (Figure 2B) of all samples supported the adequacy and rationality of the sampling efforts.

#### α Diversity Analysis and β Diversity Analysis

**FIGURE 3:**
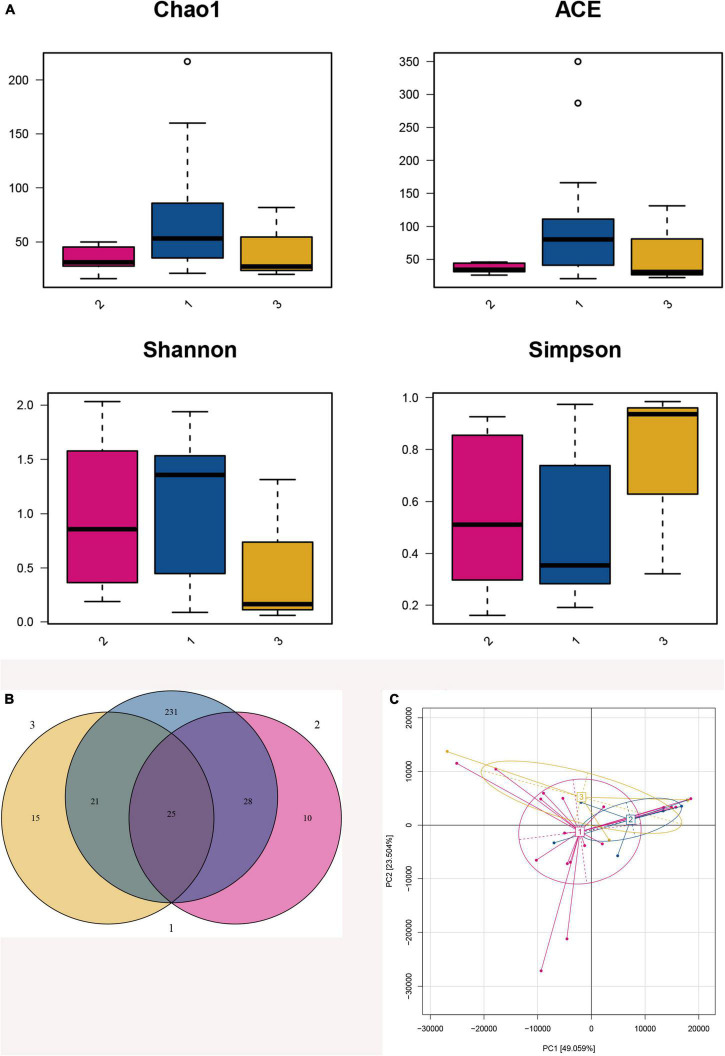
Fecal microbiota diversity and composition for 75 preterm infants. 1: The lower BMI group, 2: the higher BMI group, 3: the BMI catch-up group. **(A)** α Diversity, which was calculated with Chao1, ACE, Shannon index, and Simpson index, showed there were statistically significant differences in Chao1 and ACE index between the higher BMI group and the lower BMI group (*p* = 0.045, *p* = 0.027), indicating that the lower BMI group had more microbial types. However, there were no statistically significant differences in Shannon and Simpson index between the two groups (*p* = 0.739, *p* = 0.739), so the microbial diversity could not be considered higher in the lower BMI group. **(B)** Venn diagram of operational taxonomic units (OTUs) among the three BMI trajectory groups. **(C)** Principal component analysis was conducted based on OTU table, and showed that the gut microbiota composition in the higher BMI group, the lower BMI group, and the BMI catch-up group had a greater similarity, and it could not be significantly distinguished from the BMI growth trajectories.

α analysis among three groups found that there were no significant differences between the four indices (Chao1, ACE, Shannon, and Simpson index) (*p* > 0.05, Figure 3A). Then, we analyzed the higher BMI group and the lower BMI group and found that there were statistically significant differences in Chao1 and ACE index between the two groups (*p* < 0.05), indicating that the lower BMI group had more microbial types, and with the increase in BMI, the microbial abundance decreased. However, there were no statistically significant difference in Shannon and Simpson index between the two groups, so the microbial diversity could not be considered higher in the lower BMI group (Table 3).

**TABLE 3:** Comparisons of alpha diversity indices between the higher BMI group and the lower BMI group.

Index	Chao1	ACE	Shannon	Simpson
*p*-value	**0.045**	**0.027**	0.739	0.739

*Bold value means P < 0.05 and there was statistically significant.*

Venn diagram showed that 330 OTUs were obtained after taxonomic assignment; there were 63 OTUs in the higher BMI group, 305 OTUs in the lower BMI group, and 61 OTUs in the BMI catch-up group; 25 of these OTUs were shared among the three groups. More importantly, we found that the higher BMI group, the lower BMI group, and the BMI catch-up group had their unique OTUs, reaching10, 231, and 15, respectively. It indicated that there was a higher relative microbial abundance in the lower BMI group (Figure 3B).

Principal component analysis showed that the gut microbiota composition in the higher BMI group, the lower BMI group, and the BMI catch-up group had a greater similarity, and it could not be significantly distinguished from the BMI growth trajectories. In addition, the distribution in the BMI catch-up group was relatively separate, which indicated that the similarity of microbial composition within the group was relatively low (Figure 3C).

#### Comparison of Gut Microbiota Composition

**FIGURE 4:**
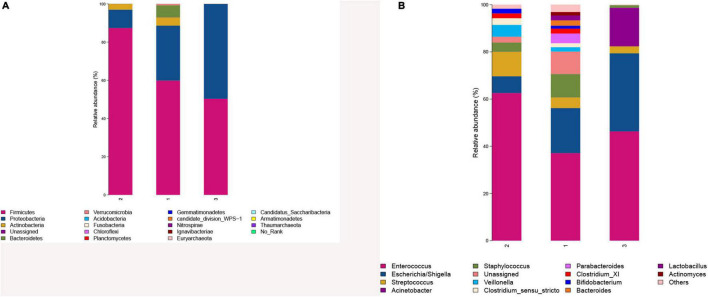
**(A)** Comparison of gut microbiota composition in the three groups at the phyla level. **(B)** Comparison of gut microbiota composition in the three groups at the genera level. 1: The lower BMI group, 2: the higher BMI group, 3: the BMI catch-up group.

Phylum identified in the three groups was mainly composed of two phyla, namely, *Firmicutes* and *Proteobacteria* (Figure 4A). The relative abundance of Firmicutes was in the order of the higher BMI group, the lower BMI group, and BMI catch-up group, whereas *Proteobacteria* was the most abundant in the BMI catch-up group and the least in the higher BMI group. At the phylum level, Metastats analysis showed that there were no significant differences in microbial abundance among the three groups, indicating that there were no significant differences at the phyla level among the three groups (Table 4).

**TABLE 4:** Differences in gut microbiota composition at the phylum level between the higher BMI group and the lower BMI group.

Phyla	Higher BMI group		Lower BMI group		*p*	*q*
		
	Mean abundance	Standard deviation	Mean abundance	Standard deviation		
*Firmicutes*	0.87	0.083	0.60	0.077	**0.032**	**0.098**
*Proteobacteria*	0.09	0.063	0.29	0.065	**0.049**	**0.146**
*Bacteroidetes*	0.00	0.000	0.06	0.047	0.149	0.447
*Actinobacteria*	0.03	0.023	0.04	0.017	0.685	0.685

*Bold value means P < 0.05 and there was statistically significant.*

Genus identified in the three groups was mainly composed of *Enterococcus* (Figure 4B). As the most abundant genus in preterm infant fecal samples in this study, *Enterococcus* relative abundance was in the order of the higher BMI group, the BMI catch-up group, and the lower BMI group. At the genus level, Metastats analysis found that there was no statistically significant difference in microbial abundance between the BMI catch-up group and the other two groups, but there was statistically significant difference in the abundance of *Lactobacillus* between the higher BMI group and the lower BMI group (*p* < 0.05), indicating that the abundance of *Lactobacillus* was lower in the higher BMI group (Table 5).

**TABLE 5:** Differences in gut microbiota composition at the genus level between the higher BMI group and the lower BMI group.

Genus	Higher BMI group		Lower BMI group		*p*	*q*
		
	Mean abundance	Standard deviation	Mean abundance	Standard deviation		
*Enterococcus*	0.63	0.131	0.37	0.074	0.061	0.184
*Escherichia/Shigella*	0.07	0.049	0.19	0.066	0.094	0.283
*Staphylococcus*	0.04	0.033	0.1	0.064	0.755	1
*Streptococcus*	0.11	0.042	0.04	0.021	0.207	0.311
*Bacillus*	0	0	0.04	0.03	0.14	0.419
*Veillonella*	0.05	0.05	0.02	0.01	0.816	0.816
*Clostridium*	0.02	0.017	0.02	0.013	0.986	0.986
*Bacteroides*	0	0	0.02	0.018	0.189	0.567
*Lactobacillus*	0	0	0.02	0.01	**0.013**	**0.040**
*Bifidobacterium*	0.02	0.02	0.01	0.007	0.898	0.898
*Actinomyces*	0	0	0.02	0.014	0.343	0.515

*Bold value means P < 0.05 and there was statistically significant.*

## Discussion

Previous studies suggested that gut microbiota can affect the growth and development of children (Durda-Masny et al., 2022) and pointed maturity of gut microbiome is necessary for long-term expected growth (Subramanian et al., 2014). In our study, the preterm infants were divided into three groups by the LCGMM: the higher BMI group, the lower BMI group, and the BMI catch-up group. The BMI growth trajectories could not be clearly distinguished because PCA showed that gut microbiota composition among these three groups was similar. The early gut microbiota of preterm infants was mainly composed of Firmicutes and Proteobacteria, and the early proportion of *Lactobacillus* was lower in the higher BMI group.

Previous studies found in the gut microbiota in human was mainly composed of six phyla, *Firmicutes, Bacteroides, Proteobacteria, Verrucomicrobia, Actinobacteria*, and *Fusobacteria*, with *Bacteroidetes* and *Firmicutes* as the dominating microbiota (Koboziev et al., 2014). Our study showed that *Firmicutes* and *Proteobacteria* composed more than 90% of the gut microbiota of preterm infants, which might be affected by the sampling age, because the change of gut microbiota in early life was a dynamic process, and tended to be stable until the age of 2 years, whereas the samples collected in the present study were within 3 months of age mostly.

A prospective cross-sectional study discovered a higher proportion of *Firmicutes/Bacteroidetes* in obese children (Bervoets et al., 2013). Riva et al. (2017) also found that obese children had higher *Firmicutes* and lower *Bacteroidetes* compared with the normal ones by analyzing gut microbiota composition of 42 obese children. Our study observed that the higher BMI group had a higher proportion of Firmicutes, but there was no statistical significance compared with the other two groups. The results of this study were different from other ones, which was due to the age, region, race, and other related factors of the subjects investigated.

Our study found that there was a significant difference in the genus abundance of *Lactobacillus* between the lower and higher BMI groups (*p* < 0.05), suggesting that the reduction of *Lactobacillus* might contribute to obesity, which was consistent with a previous study (Stanislawski et al., 2018). A previous study examined the association between gut Lactobacilli and obesity dependent on dietary patterns in children, indicating that *Lactobacillus* acted as a protective factor of obesity induced by an unhealthy diet in children (Castañeda-Márquez et al., 2020). One study showed *Lactobacillus* and *Bifidobacterium* were finally used as the candidates for antiobesity strains screening; moreover, they finally got nine strains with antiobesity ability, and all of them were *Lactobacillus* (Liang et al., 2020). *Lactobacillus* is present in the gastrointestinal tract as a type of probiotic, which can ferment carbohydrates to produce a large amount of lactic acid. It is one of the important physiological bacteria in human beings. In addition to immunity enhancement, anti-inflammation, and anticancer, it also plays an important role in the regulation of gut microbiota. Studies find that *Lactobacillus* can significantly reduce abdominal fat and lose weight in obese mice, which might be due to the fact that *Lactobacillus* can reduce fat absorption and affect energy metabolism (Kadooka et al., 2010), and *Lactobacillus* has been shown to reduce food intake to curb obesity in mice (Yun et al., 2009).

Many studies found that the abundance of gut microbiota in obese children was lower than that in normal or underweight children (Cotillard et al., 2013; Le Chatelier et al., 2013). Kong et al. (2013) found that losing weight increased gut microbial abundance in humans, indicating that the gut microbial abundance in the obese was lower than the normal, and this change could be reversed by losing weight. One study found that the abundance of gut microbiota obviously decreased when BMI fell, which were statistically significant; the diversity indices including Shannon index and Simpson index also gradually decreased, but the differences were not statistically significant (Chen et al., 2021). Our study also revealed that the abundance of gut microbiota in the lower BMI group was significantly higher than that of the higher BMI group, and there were significant differences in Chao1 and ACE between the two groups, whereas there were no significant differences between the Shannon index and Simpson index. It might be because there was a higher abundance of gut microbiota but lower evenness in the lower BMI group, so there were no significant differences in microbial diversity between the two groups.

In the present study, we found a certain correlation between the BMI growth trajectories and gut microbiota diversity, but the causal relationship needed to be further confirmed. The possible mechanisms of gut microbiota leading to high BMI levels were as follows: polysaccharides or fats that were not easily digested and absorbed by the gastrointestinal tract could be decomposed into monosaccharides or short-chain fatty acids (SCFAs) by gut microbiota, thereby increasing the energy intake of the host. The intestinal epithelial cells could be stimulated to grow by three types of SCFA: acetic acid, propionic acid, and butyric acid, so that the intestinal absorption of nutrients increased (Payne et al., 2011). SCFA could also act on G protein–coupled receptors 43 and 41 to accelerate the synthesis and secretion of glucagon peptide-1 and peptide tyrosine–tyrosine by enteroendocrine cells, which could inhibit peristalsis, prolong the passage time of intestinal contents, increase the absorption of nutrients, enhance sugar-induced insulin secretion, and eventually cause energy and fat accumulation (Duca et al., 2013). Another was that alteration of gut microbiota could damage the integrity of the intestinal epithelial barrier, change the intestinal permeability, and increase the lipopolysaccharide, a component of the cell wall of gram-negative bacteria in the intestinal tract, which entered circulation to cause an immune response, affected insulin signaling pathway, and thus promoted insulin resistance and obesity (Caricilli and Saad, 2014).

Some limitations in this study should be noticed. First, the subjects only come from Hunan Children’s Hospital, which results in a single source of subjects and few cases, some of our results may not generalize to other ethnic or racial groups or geographic regions. Second, the gut microbiota in humans tends to be stable after the age of 2 years, but most fecal samples from the preterm infants who were younger than 3 months were collected in the present study. The effects of the sampling age and the underlying disease were not considered. Third, because of the small number of preterm infants in the BMI catch-up group, it is particularly necessary to be cautious in extrapolating the results of this study. Fourth, there are plenty of researches indicating that preterm and term infants have a different composition of gut microbiota; one suggested that *Escherichia coli*, *Bacteroides*, and *Enterococcus* were the main colonizing bacteria in full-term infants, whereas *Clostridium* and facultative anaerobes were the main colonizing bacteria in preterm infants (Ferraris et al., 2012), and the other one pointed that the composition of gut microbiota in preterm infants is much higher in *Enterobacteriaceae*, *Enterococcus*, and other opportunistic pathogenic microorganisms, which differs from full-term infants (Hill et al., 2017); besides, there are some studies that showed children obesity is related to *Firmicutes, Bifidobacterium, Firmicutes-to-Bacteroidetes ratio, Lactobacillus*, and *Akkermansia* (Da Silva et al., 2020; Liang et al., 2020); therefore, the conclusions may not be extrapolated to healthy full-term infants. Finally, the present study is an observational study, and there are no interventions; moreover, we only collected stool samples from the neonatal period and did not perform dynamic monitoring of gut microbiota, so the causal relationship between the gut microbiota and high BMI level could not be established by this study.

Overall, the microbial abundance was significantly less in the higher BMI group than in the lower BMI group for preterm infants, but there were no significant differences in microbial diversity.

## Conclusion

The relative abundance of the *Lactobacillus* genus was lower in the higher BMI growth trajectory, whereas the diversity of gut microbiota was not significant differences among different BMI growth trajectories in preterm infants.

## Data Availability Statement

The raw sequence datasets from 16S rDNA gene sequencing are available in the SRA database under accession number PRJNA814846.

## Ethics Statement

The studies involving human participants were reviewed and approved by the Ethics Committee of Hunan Children’s Hospital. Written informed consent to participate in this study was provided by the participants’ legal guardian/next of kin.

## Author Contributions

JQ, QFZ, and JYY contributed to the conception or design of the work. JQ, STX, and JD performed the statistical analyses. STX and CCZ drafted the manuscript. XLL and ZHX provided critical revision of the manuscript. All authors read and gave final approval of the version to be published.

## Conflict of Interest

The authors declare that the research was conducted in the absence of any commercial or financial relationships that could be construed as a potential conflict of interest.

## Publisher’s Note

All claims expressed in this article are solely those of the authors and do not necessarily represent those of their affiliated organizations, or those of the publisher, the editors and the reviewers. Any product that may be evaluated in this article, or claim that may be made by its manufacturer, is not guaranteed or endorsed by the publisher.
